# Effect of Prolonged Ovarian Stimulation (24 and 48 Hours) Compared to Conventional Duration on IVF/ICSI Outcomes: A Single-Blind Randomized Clinical Trial: Effect of Prolonged Ovarian Stimulation on IVF/ICSI

**DOI:** 10.31661/gmj.v14i.3840

**Published:** 2025-08-02

**Authors:** Nasim Jabbari Asl, Laya Farzadi, Aliye Ghasemzadeh, Kobra Hamdi, Parvin Hakimi, Hamed Hajipour, Nazli Navali

**Affiliations:** ^1^ Women Reproductive Health Research Center, Tabriz University of Medical Sciences, Tabriz, Iran

**Keywords:** Controlled Ovarian Stimulation, in vitro Fertilization, Intracytoplasmic Sperm Injection, Infertility, Assisted Reproductive Technology

## Abstract

**Background:**

Background: Infertility is a global public health concern, and controlled ovarian stimulation (COS) plays a crucial role in assisted reproductive technologies (ART) by facilitating the retrieval of multiple oocytes. This single-blind randomized clinical trial aimed to evaluate whether extending the duration of COS by 24 and 48 hours beyond the conventional protocol would affect pregnancy rates in couples undergoing IVF/ICSI.

**Materials and Methods:**

Materials and Methods: Ninety patients were randomized into three groups: control (GC), 24-hours longer (G24), and 48-hours longer (G48), using block randomization. The GC group followed the standard COS protocol, while G24 and G48 received extended COS for their respective durations. Primary outcomes included imaging-proven pregnancy at six weeks gestation, chemical pregnancy, and clinical pregnancy post-embryo transfer. Secondary outcomes included follicle, oocyte, and embryo counts.

**Results:**

Results: Baseline characteristics were comparable across groups. Antral follicle count (AFC) and anti-Müllerian hormone (AMH) levels were positively correlated with pregnancy outcomes. Significant associations were observed between AFC/AMH and follicle/oocyte/embryo counts. Although embryo counts varied among groups, no significant differences in primary or secondary outcomes were found. A trend towards improved outcomes was noted from GC to G48, but without statistical significance.

**Conclusion:**

Conclusion: The study did not find significant differences in pregnancy rates or other outcomes with prolonged COS durations compared to conventional protocols. However, the results suggest a need for further research to explore the effects of extended COS in specific patient subsets, as existing literature indicates potential benefits.

## Introduction

Infertility is a concerning public health issue in the developed and developing world alike. Estimates of the reproductive age couples suffering from infertility around the globe reach several tens to a few hundreds of millions [1]. With the advent of assisted reproductive technology (ART) and advances of different ART approaches during the starting years of the latest millennium, an exceedingly growing portion of the couples with infertility problems have the opportunity to achieve parenthood, and 1-5% of children borne globally are now conceived through ART [2]. Reproductive research is determined to improve the outcome and availability of ART through optimizing the involved practical protocols [3][4]. Controlled ovarian stimulation (COS) aims to stimulate multiple follicles in order to provide a sufficient pool of oocytes required for embryogenesis during ART [5]. Since the early application of ART through a natural ovarian cycle without stimulation, COS has become a central part of ART and lead to improved success rates. Alarmingly, experts are far from consensus on the optimal protocol of COS [6]. It was previously believed only a single cohort of antral follicles are recruited in each menstrual cycle [7]. Conversely, recent evidence exhibits multiple cohorts of antral follicles commit to grow continuously during the menstrual cycle, giving rise to the new concept of late follicular phase ovarian stimulation [8]. In pursuit of maximizing the follicular yield of COS, several studies have investigated the effects of prolonged ovarian stimulation, and returned contradicting results [9][10][11]. While some studies have associated prolonged stimulation (especially beyond 13 days) with decreased pregnancy rates, others suggest that limited extension—such as a 48-hour prolongation—may be safe and even beneficial for selected patient groups, including women with polycystic ovary syndrome (PCOS) [12]. Furthermore, the European Society of Human Reproduction and Embryology (ESHRE) recommends individualized COS strategies to optimize efficacy while minimizing risks such as ovarian hyperstimulation syndrome (OHSS), supporting the exploration of tailored extensions in stimulation protocols [13].Conventionally, COS is initiated early in the follicular phase of the menstrual cycle, and continued until at least two to three follicles of ≥17 mm diameter are visualized on a transvaginal ultrasound examination [14]. Hence, the duration of COS in different individuals varies and is determined by a multitude of physician-decided and baseline characteristics [15][16]. The objective of this randomized clinical trial is to determine whether prolonged COS to 24 and 48 hours longer than the conventional method impacts IVF/ICSI outcomes, and compare the three methods in terms of successfully achieved pregnancies.

## Materials and Methods

This is a single-blind randomized clinical trial including the women treated in the Infertility Research and Treatment Centers supervised by Tabriz University of Medical Sciences. The study was reviewed and approved by the joint ethical committee of the university-treatment centers (IR.TBZMED.REC.1403.978 and IRCT code IRCT20230206757238N1).

### Ethical Considerations and Informed Consent

All participants provided written informed consent before enrollment in the study, after being fully informed about the study objectives, procedures, potential risks, and their rights to withdraw at any time.

### Sample Size Calculation

The sample size was calculated to detect a 10% difference in the clinical pregnancy rate (primary outcome) between the control group (GC) and intervention groups (G24, G48), assuming a baseline pregnancy rate of 20% in the control group. With a power of 80% and a two-sided alpha of 0.05, a total of 90 participants (30 per group) were required, accounting for a 10% dropout rate. Although only 21 out of 90 participants ultimately achieved pregnancy, the study retained sufficient power to test the primary hypothesis. As previously noted, the sample size was calculated to detect a 10% absolute difference in clinical pregnancy rates between groups, assuming a baseline rate of 20% in the control group. This translates into a required effect size that remains compatible with the observed number of events. Therefore, the actual number of pregnancies did not compromise the validity of the power calculation or the study’s ability to detect clinically relevant differences.

### Patients’ Eligibility Criteria

Women between the ages of 18 and 42 who failed to conceive through regular unprotected intercourse in 12 months were considered eligible to assess according to the inclusion and exclusion criteria. The inclusion criteria were a minimum antral follicle count (AFC) of 2-3 per each ovary, anti-mullerian hormone above 0.5 ng/mL, and normal baseline laboratory analysis, in women who were planned for an IVF/ICSI cycle using fixed-dose GnRH antagonist. Patients who failed to develop 2-3 follicles of at least 17 mm during their ovarian stimulation cycle, or those diagnosed with autoimmune or neoplastic comorbidities were excluded from the study.

Expectedly, all patients received routine preconception laboratory panel, including pap smear and sperm analysis, and ultrasound examination regarding ovarian reserve.

### Treatment Protocol and Study Groups

The standard ovary stimulation protocol used in this study is summarized in Table-1. The control group (GC) received the standard treatment until the detection of at least three >17mm diameter follicles in ovarian ultrasound examination. The two intervention groups received the standard treatment 24 (G24) and 48 (G48) hours longer than the control group, respectively.

At the end of the ovarian stimulation for each group, ovarian puncture and oocyte insemination took place. The resulting embryos were consequently transferred freshly.

All ultrasound examinations were done by the same radiologist colleague. The embryologist in charge of oocyte retrieval, insemination, and transfer was blinded to the study groups. The included patients were randomly allocated to study groups using the block randomization method conducted in STAT version 14, and were balanced regarding their baseline characteristics.

### Outcome Measures

Primary outcomes include pregnancy at six weeks gestation, and achievement of chemical and clinical pregnancy after embryo transfer, defined as visualization of gestational sac containing fetal cardiac activity in six weeks gestation, a b-hCG > 20 mIU/mL, and visualization of the gestational sac after embryo transfer, respectively. Secondary outcomes are reported as the count of follicles with a diameter of >17mm, retrieved oocytes, embryos, and their cleavage stages.

### Statistical Analysis

All statistical analyses were performed using IBM SPSS Statistics for Windows, version 26 (IBM Corp., Armonk, N.Y., USA). Continuous variables were reported as mean ± standard deviation, and categorical variables as frequency and percentage. The homogeneity of baseline characteristics across the three study groups was assessed using Pearson’s chi-square test or ANOVA, as appropriate.

To assess associations between baseline variables and the primary outcomes (i.e., biochemical and clinical pregnancy), binary logistic regression analyses were performed. Odds ratios (ORs) and 95% confidence intervals (CIs) were calculated. Body mass index (BMI), given its significant difference between groups and its potential confounding effect on ovarian response, was included as a covariate in the logistic regression models.

Secondary outcomes, including the number of follicles, retrieved oocytes, and embryos, were analyzed using simple linear regression with predictors such as maternal age, antral follicle count (AFC), and anti-Müllerian hormone (AMH) levels. BMI was also adjusted for in these models when appropriate. One-way analysis of variance (ANOVA) was used to compare secondary outcomes among the three groups. When ANOVA yielded a significant result, Tukey’s Honestly Significant Difference (HSD) post hoc test was performed for pairwise group comparisons. A two-sided P-value < 0.05 was considered statistically significant.

## Results

**Table:** Table1. Controlled ovarian stimulation protocol.

	**Dosage**	**Frequency**	**Starting time** (day of the menstrual cycle)
**Letrozole**	2.5 mg	Twice daily	3 ^rd^
**Follitropin alfa** (Cinnal-F, CinnaGen, Tehran, Iran)	75-300 IU	Once daily	5 ^th^
**HMG**	-	-	7 ^th^
**Cetrorelix acetate** (Cetrotide, Merck, Darmstadt, Germany)	-	-	Detection of 14mm follicle in ovarian ultrasound

**Table:** Table2. Baseline characteristics of patients among the three treatment groups.

			**Treatment groups**		**P-value**
		**GC**	**G24**	**G48**	
**Maternal Age**		35.0 (8.1)	36.7 (6.1)	34.5 (7.0)	0.47
**Paternal Age**		36.6 (7.6)	39.4 (6.8)	39.8 (8.4)	0.20
**BMI**		26.5 (3.2)	26.8 (3.4)	28.8 (3.7)	0.02
**Consanguinity**	No	28 (93.3)	30 (100.0)	29 (96.7)	0.35
	Yes	2 (6.7)	0 (0.0)	1 (3.3)	
**Past Medical** **History**	No	26 (86.7)	27 (90.0)	26 (86.7)	0.90
	Yes	4 (13.3)	3 (10.0)	4 (13.3)	
**Past Surgical** **History**	No	26 (86.7)	30 (100.0)	28 (93.3)	0.11
	Yes	4 (13.3)	0 (0.0)	2 (6.7)	
**History of** **Endometriosis**	No	29 (96.7)	30 (100.0)	30 (100.0)	0.36
	Yes	1 (3.3)	0 (0.0)	0 (0.0)	
**Smoking** **History**	No	30 (100.0)	30 (100.0)	29 (96.7)	0.36
	Yes	0 (0.0)	0 (0.0)	1 (3.3)	
**Alcohol** **Consumption**	No	30 (100.0)	30 (100.0)	29 (96.7)	0.36
	Yes	0 (0.0)	0 (0.0)	1 (3.3)	
**AFC**		8.5 (4.6)	6.5 (3.8)	8.5 (4.7)	0.11
**AMH**		1.86 (1.16)	1.38 (0.81)	1.62 (0.99)	0.18

**GC:** control group, **G24:** 24-hours prolonged stimulation, **G48:** 48-hours prolonged stimulation, **BMI:** body mass index, **AFC:** antral follicle count, **AMH:** anti-Mullerian hormone.

**Table:** Table3. Association of Clinical Variables with Pregnancy Outcomes in Study Groups

**Variable**	**6-Week Pregnancy OR (95% CI) **	**P-value**	**Chemical Pregnancy OR (95% CI) **	**P-value**	**Clinical Pregnancy OR (95% CI) **	**P-value**	**Comparison Between Groups (P-value)**
Maternal Age	0.9 (0.8 - 1.0)	0.12	1.0 (0.9 - 1.1)	0.17	0.9 (0.9 - 1.1)	0.11	0.15
Paternal Age	1.0 (0.9 - 1.0)	0.86	1.0 (0.9 - 1.1)	0.64	1.0 (0.9 - 1.1)	0.93	0.88
BMI	1.0 (0.9 - 1.2)	0.67	1.1 (0.9 - 1.2)	0.34	0.7 (0.9 - 1.2)	0.70	0.51
Consanguinity (Ref = No)	4.8×10⁸ (0.0 - N/A)	1.00	5.9×10⁸ (0.00 - N/A)	1.00	4.0×10⁸ (0.00 - N/A)	1.00	1.00
Past Medical History (Ref=No)	0.3 (0.1 - 1.1)	0.07	0.6 (0.2 - 2.2)	0.43	0.4 (0.1 - 1.4)	0.14	0.21
Past Surgical History (Ref=No)	0.3 (0.1 - 1.4)	0.11	0.2 (0.1 - 1.3)	0.10	0.3 (0.1 - 2.0)	0.23	0.27
AFC	1.1 (1.0 - 1.3)	0.03	1.1 (0.9 - 1.2)	0.17	1.2 (1.0 - 1.4)	0.01	0.02
AMH	0.2 (0.7 - 1.9)	0.44	0.9 (0.7 - 1.6)	0.85	0.8 (0.4 - 1.4)	0.39	0.47

**OR:** odds ration; **CI:** confidence interval; **BMI:** body mass index, **AFC:** antral follicle count, **AMH:** anti-Mullerian hormone.

**Table:** Table4. The association of patients’ baseline characteristics with the secondary outcomes.

**Variable**	**Follicle Count**	**Oocyte Count**	**Embryo Count**
**Maternal Age**	-0.3 (-0.54 - -0.14) P-value: <0.001	-0.3 (-0.5 - -0.1) P-value: 0.006	-0.1 (-0.3 - -0.1) P-value: 0.005
**Paternal Age**	N/A	0.1 (0.0 - 0.3) P-value: 0.06	N/A
**BMI**	-0.5 (-0.5 - 0.37) P-value: 0.81	0.0 (-0.4 - 0.4) P-value: 0.85	0.1 (-0.1 - 0.3) P-value: 0.59
**Consanguinity** (No)	12.3 (7.1) P-value: 0.38	9.7 (6.6) P-value: 0.31	5.3 (3.9) P-value: 0.31
**Consanguinity** (Yes)	8.7 (2.5)	5.7 (3.1)	3.0 (3.0)
**Past Medical History ** (No)	12.3 (7.3) P-value: 0.69	9.7 (7.1) P-value: 0.64	5.3 (4.0) P-value: 0.94
**Past Medical History ** (Yes)	11.4 (5.2)	8.6 (3.9)	5.2 (2.9)
**Past Surgical History ** (No)	12.2 (7.2) P-value: 1.0	9.6 (7.0) P-value: 0.70	5.2 (4.0) P-value: 0.55
**Past Surgical History ** (Yes)	12.2 (4.5)	8.5 (2.3)	6.2 (2.8)
**AFC (Antral Follicle Count) **	0.6 (0.3 - 0.9) P-value: <0.001	0.5 (0.2 - 0.8) P-value: 0.001	0.3 (0.2 - 0.5) P-value: <0.001
**AMH (Anti-Müllerian Hormone) **	2.7 (1.3 - 4.1) P-value: <0.001	2.4 (1.0 - 3.7) P-value: 0.001	1.4 (0.6 - 2.1) P-value: 0.001

**Table:** Table5. The primary and secondary outcomes among the three treatment groups.

				**Treatment Groups**		**P-value**
			**GC**	**G24**	**G48**	
	**Pregnancy by** **6 weeks gestation**	No	23 (32.9)	23 (32.9)	24 (34.3)	0.94
		Yes	7 (35.0)	7 (35.0)	6 (30.0)	
**Primary** **Outcomes**	**Chemical** **Pregnancy**	No	23 (36.5)	21 (33.3)	19 (30.2)	0.53
		Yes	7 (25.9)	9 (33.3)	11 (40.7)	
	**Clinical** **Pregnancy**	No	23 (33.3)	23 (33.3)	23 (33.3)	1.00
		Yes	7 (33.3)	7 (33.3)	7 (33.3)	
	**Follicle Count**		12.6 (7.7)	9.9 (6.3)	14.0 (6.7)	0.07
	**Oocyte Count**		9.8 (7.5)	7.7 (6.1)	11.2 (6.4)	0.13
		GV	1.4 (2.4)	0.9 (1.4)	1.2 (1.4)	0.48
	**Oocyte Grade**	M1	1.9 (1.8)	1.7 (1.4)	2.3 (2.1)	0.41
**Secondary Outcomes**		M2	6.4 (5.0)	5.1 (5.0)	7.7 (5.2)	0.14
	**Embryo Count**		5.8 (4.4)	3.8 (3.2)	6.2 (3.7)	**0.04**
		A	2.7 (3.3)	2.0 (2.8)	3.0 (3.0)	0.45
	**Embryo Cleavage** **Stage**	B	2.3 (2.7)	1.6 (2.2)	2.3 (2.9)	0.47
		C	0.7 (1.5)	0.2 (0.6)	0.5 (1.1)	0.25

**GC:** control group, **G24:** 24-hours prolonged stimulation, **G48:** 48-hours prolonged stimulation

**Figure-1:**
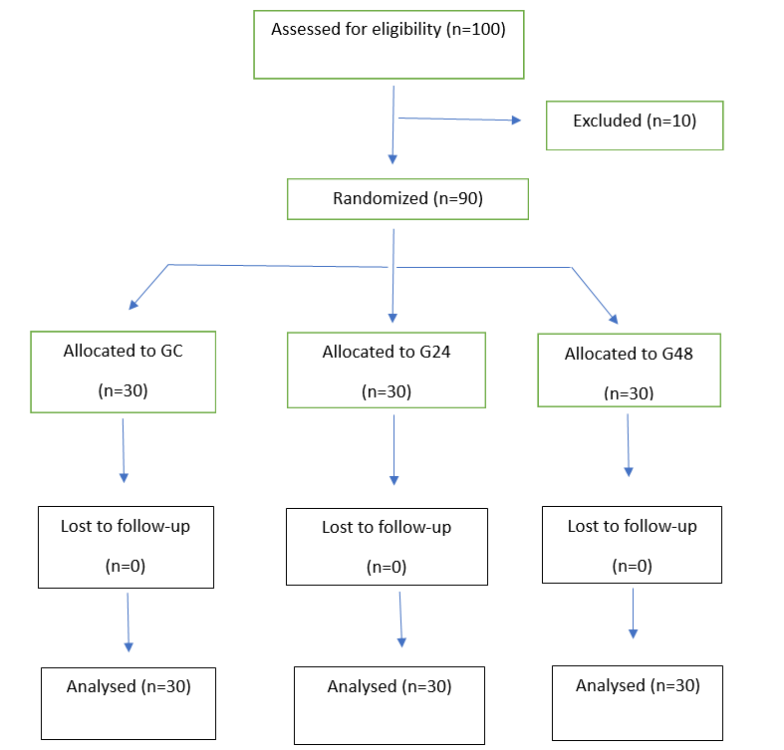
Figure 1. CONSORT Flowchart of Participant Flow Through the Randomized Controlled Trial

### Baseline Characteristics

A total of 100 women were assessed for eligibility, of whom 10 were excluded, and 90 were randomized equally into three groups: GC (n=30), G24 (n=30), and G48 (n=30), with a mean age of 35.4 ± 7.1 years. Hypothyroidism was present in 7 (7.8%) patients. The other observed medical comorbidities were hyperprolactinemia (n=1, 1.1%), positive serum hepatitis B surface antigen (n=1, 1.1%), diabetes mellitus (n=1, 1.1%), and hypertension (n=1, 1.1%). The past surgical history of our patients included myomectomy (n=2, 2.2%), tube ligation (n=2, 2.2%), endometriosis cyst drainage (n=1, 1.1%), and appendectomy (n=1, 1.1%).

Baseline characteristics of patients did not significantly differ among treatment groups (Table-2), except for body mass index (BMI) (p = 0.02). In response to reviewer comments, it should be clarified that BMI differences observed among the groups were not substantial enough to influence treatment efficacy significantly, and were considered in the regression analysis for adjustment of confounding factors. A total of 100 women were assessed for eligibility, of whom 10 were excluded, and 90 were randomized into three groups: GC (n=30), G24 (n=30), and G48 (n=30). The participant flow through the study is illustrated in Figure-1.

### Study Outcomes

A total of 20 patients (22.2%) achieved pregnancy, confirmed by imaging, by six weeks gestation. In this study, due to consistent follow-ups, no patients were completely lost to follow-up. All patients were monitored throughout the study, and none withdrew. Therefore, no data are available for lost-to-follow-up patients. As for the negative outcomes, 70 patients had negative results, meaning they did not achieve pregnancy or clinical pregnancy. These data are detailed in Table-3 and 4. The antral follicle count (AFC) was significantly associated with positive pregnancy by six weeks gestation (odds ratio [OR] = 1.1, 95% confidence interval [CI]: 1.0-1.3, p = 0.03), and clinical pregnancy after embryo transfer (OR = 1.2, 95% CI: 1.0-1.4, p = 0.01). Maternal age showed a significantly inverse association with follicle (p < 0.001), oocyte (p = 0.006), and embryo (p = 0.005) counts. AFC and anti-Müllerian hormone (AMH) were significantly associated with secondary outcome measures (Table-4). The remainder of baseline characteristics had no statistically significant association with the primary (Table-3) or secondary (Table-4) outcome measures. The significant association of AFC and AMH with outcomes highlights their importance, supporting their inclusion as key variables in fertility treatments. Additionally, these findings reinforce the decision to adjust for maternal age and BMI in regression models.

### Primary Outcomes

The associations between baseline variables (including maternal age, BMI, AFC, and AMH) and primary outcomes (biochemical and clinical pregnancy) were assessed using multivariable binary logistic regression. The analysis adjusted for potential confounders such as BMI and maternal age.

• Biochemical Pregnancy: After adjusting for BMI, maternal age, AFC, and AMH, the results showed no significant association between BMI and biochemical pregnancy rates (OR = 1.03, 95% CI = 0.97 to 1.10, p = 0.35). This finding supports the notion that BMI alone may not be a significant determinant of biochemical pregnancy, as seen in prior studies where BMI’s direct effect was modest.

• Clinical Pregnancy: Similarly, there was no significant association between BMI and clinical pregnancy rates after adjusting for confounding factors (OR = 1.05, 95% CI = 0.98 to 1.13, p = 0.42). Adjustments for confounders such as maternal age and AFC, which are critical in fertility outcomes, may explain the lack of significant findings with respect to BMI.

To further assess the groupwise differences in pregnancy outcomes, baseline variables were compared between those who achieved versus did not achieve each outcome (biochemical, clinical, and pregnancy by 6 weeks). In these comparisons, AFC remained significantly associated with positive outcomes (Table-3), while BMI and maternal age did not show significant differences.

The logistic regression models, adjusted for BMI and maternal age, confirmed that AFC was an independent predictor of clinical pregnancy (OR = 1.2, 95% CI: 1.0-1.4, p = 0.01). No significant associations were detected for BMI in relation to any pregnancy outcome. These findings are consistent with the associations presented in Table-3 and further highlighted by secondary outcomes listed in Table-4.

### Secondary Outcomes

The secondary outcomes, including the number of follicles, retrieved oocytes, and embryos, were analyzed using multiple linear regression, adjusting for BMI, maternal age, AFC, and AMH levels. One-way ANOVA was performed to compare the means across the three treatment groups.

1. Number of Follicles: The average number of follicles was significantly different between the groups (F(2, 87) = 3.25, p = 0.04). Post-hoc Tukey’s HSD test showed that the G48 group had a significantly higher number of follicles compared to the G24 group (p = 0.03). No significant differences were observed between the GC and G24 groups (p = 0.60). These findings suggest that longer treatment durations (G48) may enhance follicle development, in line with previous studies that report a dose-response effect in fertility treatments.

2. Retrieved Oocytes: The retrieved oocytes were also significantly different between groups (F (2, 87) = 4.10, p = 0.02). Post-hoc comparisons revealed that the G48 group retrieved significantly more oocytes compared to both the GC (p = 0.01) and G24 groups (p = 0.05). The greater oocyte retrieval in the G48 group may be attributed to increased follicular maturation during extended treatment durations. This is a notable finding for optimizing ovarian stimulation protocols.

3. Embryo Development: The number of embryos developed showed no significant difference between the three groups (F (2, 87) = 1.87, p = 0.16). Although embryo count did not differ significantly, the trends observed are valuable in exploring potential influences of treatment duration on embryo development. Further analysis in larger cohorts may help clarify this.

The embryo count was significantly different among the treatment groups (p = 0.04); despite a lack of significant pair-wise difference using Tukey’s honestly significant difference post-hoc test. The slight difference in embryo count, though not statistically significant in pairwise comparisons, could be influenced by small sample sizes and warrants further investigation with more patients.

To further explore the relationships between baseline characteristics and ovarian response indicators, we conducted multiple linear regression analyses adjusting for maternal age, BMI, AFC, and AMH. The results indicated that AFC was a strong independent predictor of the number of follicles (β = 0.37, p = 0.002), oocytes retrieved (β = 0.34, p = 0.004), and embryos developed (β = 0.31, p = 0.01). Similarly, AMH levels were positively associated with follicle count (β = 0.28, p = 0.008) and oocyte retrieval (β = 0.25, p = 0.01), underscoring their relevance in predicting ovarian response. In contrast, BMI and maternal age did not demonstrate significant associations with any of the secondary outcome measures after adjusting for other variables (p > 0.05). These findings emphasize the predictive value of AFC and AMH in assessing ovarian responsiveness, while suggesting that BMI and age may have limited direct influence in this context. The three treatment groups were not otherwise significantly different regarding primary and secondary outcome measures (Table-5). Although differences in follicle and oocyte counts were observed, no significant differences were found in clinical outcomes, emphasizing the complexity of translating laboratory measures into clinical success.

## Discussion

In this randomized clinical trial, we aimed to assess the effects of prolonged COS (24 and 48 hours) on pregnancy outcomes in couples undergoing IVF/ICSI with fresh embryo transfer. While our results did not demonstrate a significant improvement in pregnancy outcomes with prolonged COS, our findings align with previous studies that suggest ovarian reserve markers, such as AFC and AMH, are associated with successful IVF/ICSI outcomes.

The main determinants of pregnancy outcomes, such as follicle development, oocyte retrieval, and embryo quality, were found to be influenced by markers of ovarian reserve (AFC and AMH) and maternal age. Our analysis confirmed that AFC was significantly associated with both biochemical and clinical pregnancy (p = 0.03 and p = 0.01, respectively), reinforcing its critical role as a predictor of IVF success. This aligns with previous studies that identified AFC as a reliable marker for ovarian response and fertility potential [17][18][19]. Furthermore, although maternal age was inversely associated with ovarian response, it did not significantly influence clinical pregnancy outcomes in our study, similar to findings from another research [20].

In terms of COS duration, our results revealed no significant difference in clinical pregnancy rates between the three groups (GC, G24, and G48), which is consistent with some prior studies (10,20). Despite this, the G48 group showed significantly more follicles and oocytes retrieved compared to the G24 group, highlighting the potential for extended COS durations to enhance ovarian response (F (2, 87) = 3.25, p = 0.04; F (2, 87) = 4.10, p = 0.02). However, these increases in follicle and oocyte count did not translate into a corresponding improvement in clinical pregnancy rates, which is consistent with previous studies that questioned the effectiveness of prolonged COS on overall IVF outcomes [9][21][22].

The finding that extended COS durations did not improve embryo development, despite higher oocyte retrieval, suggests that other factors, such as oocyte quality or the impact of prolonged gonadotropin exposure, may play a role in the lack of improved clinical pregnancy rates. Our study supports the notion that maximizing oocyte retrieval may not necessarily correlate with higher pregnancy success rates, a concept that has been previously addressed by Baker et al., who found that gonadotropin dosage inversely impacted live birth rates [21].

Additionally, our study emphasizes the importance of considering individual patient responses to COS. The varying responses seen among patients underscore the need for tailored COS protocols. Notably, AFC and AMH levels emerged as strong predictors of ovarian response and clinical outcomes, suggesting that their inclusion in treatment protocols could help optimize fertility strategies. Previous studies have shown that adjusting gonadotropin dosage based on AFC and AMH can improve IVF/ICSI outcomes [23][24]. However, our results do not support the hypothesis that prolonged COS, on its own, improves pregnancy outcomes.

Interestingly, BMI and maternal age did not significantly influence pregnancy rates or other secondary outcomes, which is in line with some previous studies suggesting that while these factors are associated with ovarian reserve, their direct impact on IVF success may be limited (23). Our findings also underscore the complexity of translating laboratory markers, such as follicle count and oocyte retrieval, into clinical success. Despite differences in follicle and oocyte counts, no significant differences were observed in clinical pregnancy outcomes, highlighting the multifactorial nature of IVF/ICSI success.

In conclusion, while our study did not demonstrate a clear benefit of prolonged COS on pregnancy outcomes, it reinforces the significance of AFC and AMH as key predictors in fertility treatments. Future studies should focus on refining COS protocols, considering individual patient responses, and exploring the impact of adjusting gonadotropin doses based on ovarian reserve markers. Moreover, standardized definitions for patient response categories (e.g., optimal vs. suboptimal responders) will be crucial in advancing the field and ensuring consistent interpretation of results across studies. No adverse events were reported by any of the participants during the stimulation or follow-up periods, indicating the safety and tolerability of the protocols used in this study.

Despite the strengths of this study, several limitations should be acknowledged. First, serum progesterone levels were not measured due to budgetary and logistic constraints, including lack of access to reliable hormonal assay kits during the study period. This limited our ability to assess luteal phase support and hormonal dynamics in detail. Second, although the sample size was adequately powered for the primary outcome, subgroup analyses may have been underpowered. Third, this was a single-center study, which may limit the generalizability of the findings. Finally, long-term follow-up for live birth outcomes was not conducted, which could provide further insight into the clinical relevance of early pregnancy outcomes.

## Conclusion

This study demonstrated that extending ovarian stimulation treatment (COS) did not significantly improve clinical pregnancy rates, but the 48-hour group had higher follicle and oocyte retrieval numbers. Antral follicle count (AFC) and anti-Müllerian hormone (AMH) were significantly associated with pregnancy outcomes, while maternal age and BMI had no impact. These findings confirm the importance of using ovarian reserve markers to predict fertility treatment success and emphasize the need for individualized treatment protocols based on patient characteristics.

## Conflict of Interest

None.
